# Group- and Genotype-Specific Neutralizing Antibody Responses Against Respiratory Syncytial Virus in Infants and Young Children With Severe Pneumonia

**DOI:** 10.1093/infdis/jis700

**Published:** 2012-11-21

**Authors:** Charles J. Sande, Martin N. Mutunga, Graham F. Medley, Patricia A. Cane, D. James Nokes

**Affiliations:** 1 KEMRI–Wellcome Trust Research Programme, Kilifi, Kenya; 2 Health Protection Agency, London; 3 School of Life Sciences, University of Warwick, Coventry, United Kingdom

**Keywords:** respiratory syncytial virus, neutralizing antibody, immunity

## Abstract

The effect of genetic variation on the neutralizing antibody response to respiratory syncytial virus (RSV) is poorly understood. In this study, acute- and convalescent-phase sera were evaluated against different RSV strains. The proportion of individuals with homologous seroconversion was greater than that among individuals with heterologous seroconversion among those infected with RSV group A (50% vs 12.5%; *P* = .0005) or RSV group B (40% vs 8%; *P* = .008). Seroconversion to BA genotype or non-BA genotype test viruses was similar among individuals infected with non-BA virus (35% vs 50%; *P* = .4) or BA virus (50% vs 65%; *P* = .4). The RSV neutralizing response is group specific. The BA-associated genetic change did not confer an ability to escape neutralizing responses to previous non-BA viruses.

Respiratory syncytial virus (RSV) is the most important cause of viral acute lower respiratory tract infection in young children worldwide [1]. Two distinct antigenic groups, A and B, have been identified on the basis of reaction with monoclonal antibodies and nucleotide sequence data [2, 3]. Epitopes on the fusion (F) and attachment (G) glycoproteins are the targets of neutralizing antibodies [4], which correlate well with resistance to infection [5] and disease [6]. There is some evidence of population-level interaction between RSV A and B [7], suggesting temporal variations in population-level group-specific immunity.

In addition to group-specific differences, the RSV G gene is known to undergo molecular evolution characterized by progressive accumulation of amino acid changes at an estimated rate of 0.25% of amino acids per year over the length of the protein [8]. When considered together with evidence of the greater rate of nonsynonymous-to-synonymous nucleotide substitutions [8], as well as the existence of positively selected sites on the attachment proteins of both RSV A and B [9], it is reasonable to speculate that changes within the G protein are immune driven. Recently, the emergence of a novel strain of RSV B with a 60-nucleotide duplication in the variable region of the G gene has been described (the BA genotype) [10]. Since it was first reported about 10 years ago, the BA genotype has progressed from relative novelty to becoming the most dominant genotype of RSV B globally [11]. The factors that underpin its remarkable epidemiological success have so far not been described. We hypothesized that the BA genetic change conferred a neutralization-resistance phenotype that permits BA genotype strains to escape previous host immunity, allowing for increased transmissibility in susceptible populations.

In the present study, we investigated RSV group–specific responses to both contemporary and historical test viruses, as well as the role of the recent BA genetic change in abrogating neutralizing responses generated against wild-type group B strains that did not have the duplication.

## MATERIALS AND METHODS

### Patients, Samples, and Gene Sequencing

Nasal washings were obtained from children admitted to Kilifi District Hospital with severe or very severe pneumonia, for whom RSV infection was diagnosed on the basis of immunofluorescent antibody test results (Millipore). Multiplex reverse transcription polymerase chain reaction was used to determine whether the infecting virus was from group A or B [12]. An acute-phase serum sample was collected from all children at admission, and a convalescent-phase serum sample was obtained from RSV-positive patients approximately 4 weeks later. Ethics approval for the study was granted by the Kenya Medical Research Institute Ethical Review Committee. Further details about the study population, sampling procedures, diagnostic methods, and clinical findings have been published elsewhere [13]. Details on the age and sex distribution of study participants are shown in Supplementary Table 1.

The study used the following test viruses: A2 (RSV A; isolated in Australia in 1961), Kil/A/2006 (RSV A; Kenya, 2006), 8/60 (RSV B; Sweden, 1960), and Kil/B/2008 (RSV B; Kenya, 2008). Of the 2 RSV B test viruses, 8/60 did not have the 60-nucleotide G gene duplication, while Kil/B/2008 did. The G genes of infecting viruses were sequenced between nucleotide 284 on the G gene and nucleotide 9 on the F gene (GenBank accession numbers JX453211–JX453270), while their F genes were sequenced between nucleotides 121 and 918 of the F gene (GenBank accession numbers JX453271–JX453330).

### Plaque Assay and Microplaque Reduction and Neutralization Assay

Virus titers were determined by plaque assay. Ten-fold dilutions of test virus were made in minimum essential medium (MEM) and inoculated onto HEp-2 cultures for 48 hours in 96-well plates. Cells were fixed in methanol, washed, and incubated at room temperature with a primary mouse anti-RSV immunoglobulin G (IgG) monoclonal antibody (Leica Microsystems), followed by a secondary horseradish peroxidase–linked rabbit anti mouse IgG (Dako, Denmark). Plaques were developed using aminoethylcarbazole. An enzyme-linked immunosorbent spot reader was used to count the number of plaques in each well. The plaque-reduction neutralization assay was performed by preparing serial 2-fold dilutions of sera in MEM. Fifty plaque-forming units of test virus were added to each dilution, and after incubation for 1 hour at room temperature, the material undergoing the neutralization reaction was inoculated onto HEp-2 cells and incubated at 37°C for 48 hours. Plaque development and enumeration were done as described above. Neutralizing antibody titers were calculated as the 50% neutralizing dose, using the Spearman-Karber method [14], and were expressed as plaque-reduction neutralization titers. These titers were normalized using log_10_ transformation, for statistical analyses. Seroconversion was defined as a ≥4-fold rise in the neutralizing antibody titer between the acute and convalescent phases of infection.

### Study Design and Statistical Analyses

Two arms of the study were designed to measure group- and strain-specific neutralizing antibody responses. In the first arm, serum neutralizing antibody responses to contemporary and historical group A and B test viruses were measured in the sera of infants naturally infected with contemporary RSV A and B. In the second arm of the study, 2 separate groups of children who were naturally infected with wild-type BA or non-BA viruses were used to investigate the effect of the 60-nucleotide duplication on the neutralizing response.

Data analyses were done using Stata (version 11.1; StataCorp). Group- and cross-specific neutralizing antibody responses in convalescent-phase sera were analyzed using a multilevel modeling approach. Random individual-level effects were estimated using a 1-level random-effects multiple linear regression model. In this model, convalescent-stage titers were the dependent variable, while acute-stage titers, type of response (homologous/heterologous), and age were independent variables. Comparison of homologous and heterologous responses in different age classes was done by use of a linear regression model in which the dependent variable was the log-transformed rise in titer, and the independent variables were the test and infecting viruses. Proportions seroconverting to homologous and heterologous virus were compared using the McNemar χ^2^ test.

## RESULTS

### Group Specificity of the RSV Neutralizing Response

**Figure 1.**
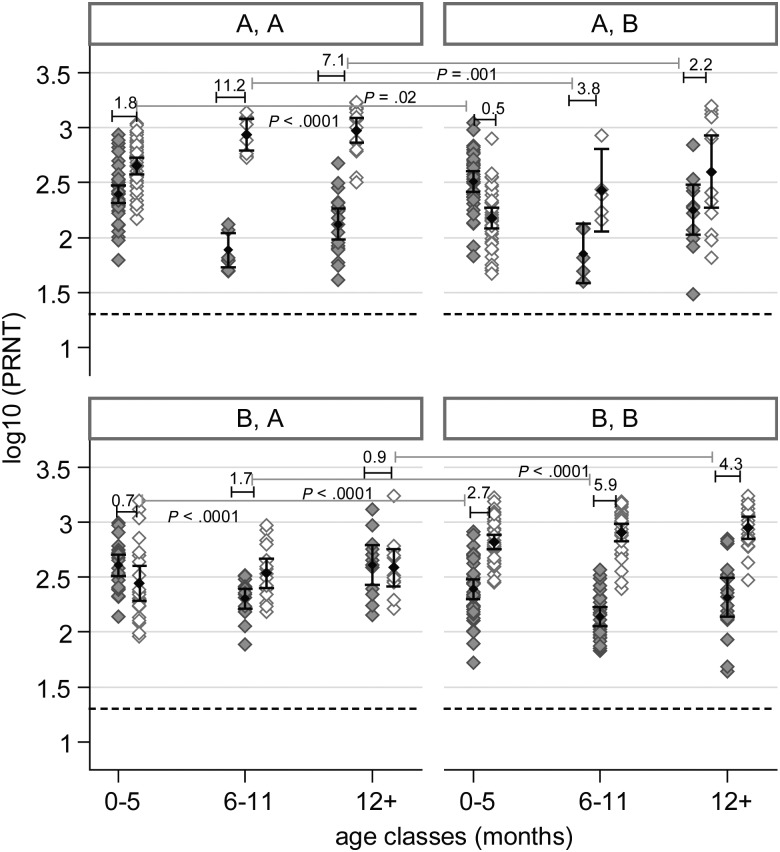
Comparison of the magnitude of the homologous and heterologous neutralizing response to both RSV A and B. The first letter in each panel heading denotes the group designation of the infecting virus, while the second letter denotes the group designation of the test virus. The grey diamond markers indicate the distribution of the acute-phase response and their corresponding means and 95% confidence intervals, while the open markers denote the distribution of convalescent-phase responses. The number above each acute/convalescent-phase pair denotes the mean fold-rise in titer from acute to convalescent phases of infection. Comparison of the magnitude of response (in terms of fold-rise in titer) to homologous virus and heterologous virus is shown by the long bars traversing the panels. The *P* value denotes whether the difference between the homologous and heterologous response in a particular age class is statistically significant. The dashed line indicates the lower limit of detection of neutralizing antibodies in this assay (defined as a plaque-reduction neutralization titer [PRNT] of <20)

Comparison of homologous and heterologous neutralizing antibody responses in different age classes was performed by testing the difference between homologous and heterologous fold-rises in titer. Results of regression analysis showed that the mean homologous response to RSV A by RSV A–infected individuals was significantly greater than their heterologous response to RSV B in the 0–5-month age class (1.8-fold vs 0.5-fold rise in titer; *P* < .0001), the 6–11-month age class (11.2-fold vs 3.8-fold rise in titer; *P* = .002), and the ≥12-month age class (7.1-fold vs 2.2-fold rise in titer; *P* = .001). Similarly, the homologous response to RSV B by RSV B–infected individuals was significantly greater than their heterologous response to RSV A in the 0–5-month age class (2.7-fold vs 0.7-fold rise in titer; *P* < .0001), the 6–11-month age class (5.9-fold vs 1.7-fold rise in titer; *P* < .0001), and the ≥12-month age class (4.3-fold vs 0.9-fold rise in titer; *P* < .0001). These data are shown in Figure 1. Group homologous and heterologous responses were further classified in terms of the ability to seroconvert. As shown in Table 1, the proportion of individuals with homologous seroconversion to both RSV A and B was significantly greater than the proportion with heterologous seroconversion. Analysis of genetic similarity between infecting viruses was done using partial F and G gene sequences. In concurrence with previous reports, there was a high level of sequence diversity on the G gene and a high level of sequence conservation on the F gene, as shown in Supplementary Table 2.

**Table 1.** Proportion of Infants Infected With Different Strains of Respiratory Syncytial Virus (RSV) Who Seroconverted to Different Test Viruses

	Test viruses % of Seroconverted Infants		
Infecting viruses	Kil/A/2006	Kil/B/2008	*P*^a^
Group A (n = 32)	50	12.5	.0005
Group B (n = 25)	8	40	.008
	A2	8/60	
Group A (n = 18)	28	0	.06
Group B (n = 20)	10	65	.001
	Kil/B/2008	8/60	
BA genotype (n = 20)	50	65	.4
Non-BA genotype (n = 20)	35	50	.4
Both genotypes (n = 40)	43	58	.1
	Kil/A/2006	A2	
Group A (n = 33)	51.5	39.4	.13
	Kil/B/2008 (+C’)	Kil/B/2008 (−C’)	
BA genotype (n = 10)	50	30	.32
Non-BA genotype (n = 10)	40	30	.32
	8/60 (+C’)	8/60(−C’)	
BA genotype (n = 10)	50	60	.16
Non-BA genotype (n = 10)	40	40	1

^a^ By the McNemar χ^2^ test, comparing differences in proportions of infants who seroconverted.

### Effect of the BA Genetic Change and Temporal Evolution on the RSV Neutralizing Response

There was no significant difference between the magnitude of the neutralizing response mounted by infants infected with non-BA strains to the 8/60 strain (3.7-fold rise in titer) and the Kil/B/2008 strain (3.42-fold rise in titer; *P* = .78). There was also no significant difference between the magnitude of the neutralizing response mounted by infants infected with BA strains to the 8/60 strain (5.13-fold rise in titer) and the Kil/B/2008 strain (3.54-fold rise in titer; *P* = .1). As shown in Table 1, no differences were found in terms of the ability to seroconvert to these 2 test viruses by either group. The effect of complement on G-specific neutralizing antibodies was investigated in a subset of infants from either group. The data presented in Table 1 show that even with the addition of complement, the proportion of infants who seroconverted to either test virus was similar, irrespective of whether the infecting group B strain contained the 60-nucleotide duplication. The effect of cumulative genetic change over approximately 45 years of RSV A evolution was tested using the sera of 33 RSV A–infected individuals. As shown in Table 1, there was no difference in the proportion who seroconverted to the A2 (1961) and Kil/A/2006 (2006) test viruses.

## DISCUSSION

The results presented in this study show that the infant serum neutralizing response to RSV is significantly group specific. Homologous seroconversion rates were significantly greater than heterologous seroconversion rates for both RSV A and B. This pattern of homologous versus heterologous reactivity was similar irrespective of whether the test viruses were contemporary or historical. Analysis of the magnitude of homologous and heterologous neutralizing responses to RSV A and B at different ages showed that homologous responses were of significantly greater magnitude than heterologous responses, irrespective of age. In this study, we were unable to definitively characterize the group specificity of the RSV neutralizing response following secondary exposure, since there were only 7 children who were >2 years old (6 with RSV A and 1 with RSV B) and who could therefore be presumed to have been undergoing secondary infection. As a result, we were unable to determine whether the pattern of responses reported here remain imprinted on secondary exposure. The data presented support the idea that sequential alternation in the transmission of RSV A and B could be the result of population-level group-specific immunity. They further provide the basis to assert that the benefit of vaccination may be enhanced if representative strains from both RSV A and B are included in future vaccines. The data also show that, despite evidence of progressive evolution over 40–50 years, the RSV neutralizing response was not altered, suggesting that future RSV vaccines may retain effectiveness over long periods, without the need for repeated antigenic updates.

Analysis of the neutralizing responses of infants who underwent natural infection with group B strains that did not contain the 60-nucleotide duplication showed that their neutralizing responses to the 8/60 strain were no different from their responses to the Kil/B/2008 strain. The proportion who seroconverted to the 8/60 strain was not statistically different from the proportion who seroconverted to the Kil/B/2008 strain, suggesting that the BA mutation does not confer the ability to escape the neutralizing responses to non-BA variants. It is possible that the neutralizing responses reported here may have been predominantly directed at the more conserved F protein or, alternatively, at the conserved region of the G protein, thus masking strain-specific responses directed at the variable parts of the G protein. Previous work has shown that G protein–specific neutralizing responses are enhanced in the presence of complement [15], suggesting that the inability to detect a difference in neutralization could potentially be attributed to this fact. To address this concern, the neutralization assays were repeated using sera from a set of infants with wild-type BA and non-BA infections. The incorporation of complement in the neutralization assays did not alter the pattern of reactivity toward the test viruses. Overall, the results suggest that the increased prevalence of the BA genotype is not accounted for by a lower susceptibility to neutralization as measured in serum antibody to non-BA variants, and the basis for the success of this new variant remains to be explained.

## Supplementary Data

Supplementary materials are available at *The Journal of Infectious Diseases* online (http://jid.oxfordjournals.org/). Supplementary materials consist of data provided by the author that are published to benefit the reader. The posted materials are not copyedited. The contents of all supplementary data are the sole responsibility of the authors. Questions or messages regarding errors should be addressed to the author.

Supplementary Data
