# Supplementary material for: Group- and Genotype-Specific Neutralizing Antibody Responses Against Respiratory Syncytial Virus in Infants and Young Children With Severe Pneumonia
Source: J Infect Dis. 2012 Nov 21;207(3):489–92. doi: 10.1093/infdis/jis700 (PMC3541697; doi:10.1093/infdis/jis700)
Supplement: Supplementary Data [file supp_207_3_489__index.html]

Group and genotype specific neutralising antibody responses against respiratory syncytial virus (RSV) in infants and young children with severe pneumonia — Group- and Genotype-Specific Neutralizing Antibody Responses Against Respiratory Syncytial Virus in Infants and Young Children With Severe Pneumonia — Group- and Genotype-Specific Neutralizing Antibody Responses Against Respiratory Syncytial Virus in Infants and Young Children With Severe Pneumonia — Supplementary Data 

# Group- and Genotype-Specific Neutralizing Antibody Responses Against Respiratory Syncytial Virus in Infants and Young Children With Severe Pneumonia

## Supplementary Data

Supplementary Data

**Files in this Data Supplement:**

- Supplementary Data - Doc file
