# Supplementary material for: Group- and Genotype-Specific Neutralizing Antibody Responses Against Respiratory Syncytial Virus in Infants and Young Children With Severe Pneumonia
Source: J Infect Dis. 2012 Nov 21;207(3):489–92. doi: 10.1093/infdis/jis700 (PMC3541697; doi:10.1093/infdis/jis700)
Supplement: Supplementary Data [file supp_jis700_jis700supp.doc]

| sex | age class (months) | | | | Total |
| --- | --- | --- | --- | --- | --- |
|  | **0-5** | **6-11** | **12-23** | **24+** |  |
| female | 19 | 6 | 6 | 5 | 36 |
| male | 31 | 15 | 8 | 2 | 56 |
| Total | 50 | 21 | 14 | 7 | 92 |

Supplementary table 1

| Comparison  Groups (n) | Amino acid identity (%) | | Nucleotide identity (%) | |
| --- | --- | --- | --- | --- |
|  | **F protein** | **G protein** | **F gene** | **G gene** |
| RSV A & B (60) | 87.6 | 28.6 | 78.7 | 45.2 |
| RSV A only (20) | 98.1 | 91.4 | 96.8 | 94.8 |
| RSV B only  (BA & non BA) (40) | 97.1 | 73.8 | 95.1 | 79.7 |
| BA only (20) | 98.6 | 93.8 | 98 | 95.2 |
| Non BA only (20) | 98.6 | 94.3 | 99.5 | 97.6 |

Supplementary table 2
